# Agreement Between TDK Silmee W22 and ActiGraph wGT3X-BT for Estimating Daily Step Counts and Moderate to Vigorous Physical Activity in Free-Living Adults: Comparative Study

**DOI:** 10.2196/64602

**Published:** 2025-07-30

**Authors:** Hiroko Shimura, Shinpei Okada, Kazushi Maruo, Kaori Daimaru, Naoki Deguchi, Yoshinori Fujiwara, Hiroyuki Sasai

**Affiliations:** 1 Research Team for Promoting Independence and Mental Health, Tokyo Metropolitan Institute for Geriatrics and Gerontology, Tokyo, Japan; 2 Department of Liberal Arts, Tokyo University of Technology, 1404-1 Katakura, Hachioji, Tokyo, 192-0982, Japan, 81 42-637-2084; 3 Physical Education and Medicine Research Foundation, Nagano, Japan; 4 Department of Preventive Medicine and Public Health, Tokyo Medical University, Tokyo, Japan; 5 Department of Biostatistics, Institute of Medicine, University of Tsukuba, Ibaraki, Japan; 6 Tokyo Metropolitan Institute for Geriatrics and Gerontology, Tokyo, Japan

**Keywords:** wearable, internet of things, activity tracker, mHealth, intensive longitudinal data, physical activity, accelerometry, ambulatory monitoring, mobile phone

## Abstract

**Background:**

Wearable Internet of Things (IoT) devices are powerful tools for remotely collecting intensive longitudinal data. The TDK Silmee W22, a wristband-type wearable IoT device with a built-in 3-axis acceleration sensor, provides minute-by-minute physical activity data such as estimated metabolic equivalents (METs) and step counts. These measurements can be aggregated to daily estimates; however, their accuracies have not been fully explored in adults under free-living settings.

**Objective:**

This study aims to assess the agreement between the TDK Silmee W22 and the research-grade activity monitor, ActiGraph wGT3X-BT, in estimating daily steps and time spent in moderate to vigorous physical activity (MVPA ≥3 METs) in adults under free-living settings.

**Methods:**

A convenience sample of young to older adults was recruited from communities across several prefectures in Japan. Participants concurrently wore a TDK Silmee W22 on their nondominant wrist and an ActiGraph wGT3X-BT on the left side of the waist during waking hours for 7 consecutive days. Data were aggregated to daily steps and time spent in MVPA (≥1952 vertical axis counts/minute for ActiGraph) for each participant. A valid day was defined as having ≥ 10 hours of accumulated ActiGraph wear time with ≥100 and <50,000 accumulated steps from both devices. Each valid day was classified as either an active day (≥10,000 steps/day or ≥21.4 minutes MVPA per day) or an inactive day. Bland-Altman plots combined with multilevel analysis and κ statistics were used to assess the agreement between physical activity estimates from the devices.

**Results:**

A total of 129 participants (n=66, 51.2% women) aged 23‐89 years provided the final dataset of 884 observations (5 to 7 daily observations/participant). The TDK Silmee W22 estimated an overall mean of 6369 (SE 242) steps/day and 40.3 (SE 1.9) minutes/day spent in MVPA. Although Bland-Altman plots suggested no obvious proportional bias, fixed biases were observed; the TDK Silmee W22 estimated −1203 steps/day (95% limits of agreement [LoA] −4202 to 1796) and +5 minutes/day (LoA−23 to 34) spent in MVPA compared with those estimated by the ActiGraph wGT3X-BT. TDK Silmee W22 and ActiGraph wGT3X-BT, respectively, classified 14% and 23.1% as active by daily step counts (κ=0.65, 95% CI 0.59‐0.72), and 70.4% and 60.9% as active by daily time spent in MVPA (κ=0.64, 95% CI 0.59‐0.69), both indicating moderate agreement.

**Conclusions:**

TDK Silmee W22 underestimated step counts and overestimated time spent in MVPA compared with the research-grade ActiGraph wGT3X-BT, which may lead to misclassification of active and inactive days. Caution is warranted when using TDK Silmee W22 data over relatively short periods, as discrepancies—particularly when compared with research-grade monitors—may affect feedback or goal setting.

## Introduction

Wearable, accelerometer-based activity monitors have been used to assess physical behavior such as physical activity, sedentary behavior, and sleep in free-living conditions [1]. The embedded sensors can monitor an individual’s behavior objectively in noninvasive and nonintrusive ways without interrupting their daily activities. Such monitors typically provide time-stamped data, which allow researchers to investigate within-individual fluctuations (eg, day-to-day changes) over time.

Over the past decade, wristband-type wearable activity trackers (eg, Fitbit, Garmin, and Xiaomi) have become increasingly popular among consumers, and there has been growing interest in using these devices for research [2,3]. These devices were originally designed to motivate individuals to increase their physical activity, which is of public health interest due to the well-documented benefits of physical activity on health [4,5]. Based on the measured triaxial acceleration signals, these activity trackers use the manufacturers’ proprietary algorithms to generate behavioral data such as step count, energy expenditure, and sleep time, allowing users to track their daily behaviors [3]. In addition, embedded rechargeable batteries and charging by the wearers themselves enable long-term monitoring, while Internet of Things (IoT) technologies facilitate automatic data upload to cloud storage through a gateway [6,7]. These features make it possible for researchers to remotely collect intensive longitudinal data [8], which consist of repeated observations over time to capture dynamic processes and individual trajectories.

**Figure 1.**
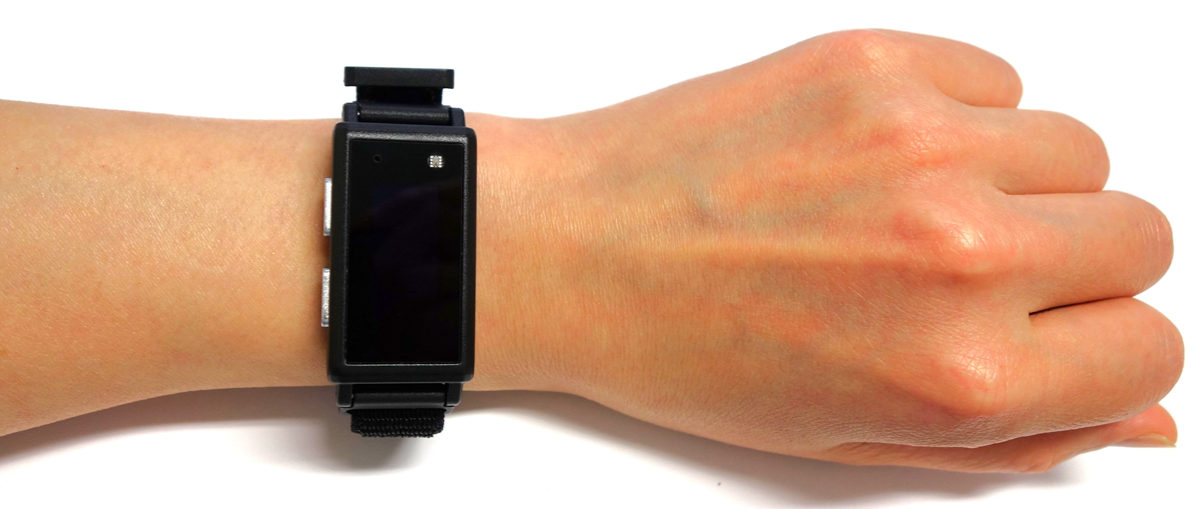
TDK Silmee W22.

The TDK Silmee W22 (TDKsw; TDK Corporation; see Figure 1) is a wristband-type wearable IoT device intended for use mainly in medical and health care services [9]. It contains multiple sensors (eg, acceleration, photoplethysmography, and skin temperature sensors), a microcontroller for analog-to-digital conversion, flash memory for storage, and a Bluetooth chip for communication, and provides behavioral data such as metabolic equivalents (METs), step count, sleep state, pulse rate, and skin temperature [9]. Data can be transferred from TDKsw to cloud storage via a gateway (smartphone or dedicated hardware), and the device is expected to be used in large-scale epidemiological studies or for delivering just-in-time adaptive interventions [10] to promote physical activity in a manner similar to other wearable IoT devices.

However, to date, only 1 study has used the TDKsw to assess physical activity [11], possibly due to insufficient examination of the accuracy of the output measures. Regarding step count, although a previous study reported a significant correlation between video observation and the TDKsw, the data were collected under a short-duration protocol (9 minutes of walking) with a limited sample of older adults [11]. Also, to our knowledge, the accuracy of the estimated METs values remains unclear. Furthermore, whereas the evaluation of the device under daily life conditions is particularly important because the results are more likely to reflect day-to-day activity, no published study has investigated TDKsw accuracy under free-living conditions.

Given these considerations, this study examined the accuracy of the TDKsw in estimating measures of physical activity using day-to-day data obtained from young to older adults in their daily lives. Daily accumulation of step counts and time spent in moderate to vigorous physical activity (MVPA ≥3 METs) were the measures examined. As a reference monitor, we chose the ActiGraph wGT3X-BT (AG; ActiGraph LLC). ActiGraph monitors have been the most frequently used to assess physical activity, accounting for approximately 50% of published studies [12], and a previous model in the same product line, the GT3X+, has been used in large-scale surveillance studies such as the US National Health and Nutrition Examination Survey, supporting the credibility and wide acceptance of this product line. This study aimed to assess the agreement between the TDKsw and AG in estimating the daily accumulation of step counts and time spent in MVPA in adults under free-living conditions.

## Methods

### Participants

A convenience sample was recruited by word of mouth through friends and colleagues of the authors (H Shimura and SO) from communities across several prefectures in Japan, including both the National Capital Region (Tokyo, Kanagawa, Saitama, and Yamanashi) and other regions (Nagano and Gifu). Eligibility criteria included age between 20 and 89 years, no gait abnormalities, having one’s own smartphone, and willingness to wear activity monitors during the monitoring period. Participants aged 70 years and older were recruited from the National Capital Region, while those aged 70 years and younger were recruited from both the National Capital Region and other regions. Efforts were made to achieve a balanced distribution of participants across 10-year age groups (20s to 80s), and to recruit men and women in roughly similar numbers within each age group wherever possible.

### Ethical Considerations

Participants who attended the introductory session were given a full explanation of the purpose and potential risks of the study. They signed an institutionally approved informed consent form before data collection. This study was conducted after obtaining approval from the institutional review board of the Tokyo Metropolitan Institute for Geriatrics and Gerontology (R22-013). It was part of the Smart Watch Innovation for Next Geriatrics and Gerontology (SWING) study, an intensive longitudinal study designed to investigate the use of behavioral data, such as physical activity and sleep monitored by wearable devices, to promote health among older adults.

### Data Collection

#### Schedule

Data were collected for approximately 1 week between January and May 2023; the specific data-collection period differed depending on the participant. At the introductory session (day 0), participants completed questionnaires regarding their demographic and anthropometric characteristics. In addition, they started to wear the TDKsw (firmware version 1.F.18) and the AG (firmware version 1.9.2). The Silmee Connect app (version 1.1.2 for iOS and version 1.1.3 for Android; TDK Corporation) was installed on each participant’s smartphone, after which their smartphone was paired with their TDKsw. They were instructed to wear both TDKsw and AG simultaneously during waking hours until day 7 and return the devices to the research team via postal mail using a prepaid return envelope on day 8.

After returning the devices, participants received a ¥5000 (approximately US $36) gift card as compensation for their time and effort in the study. This compensation was provided regardless of the amount of data collected to ensure voluntary participation.

#### Demographic and Anthropometric Characteristics

Age, gender, living arrangement (“living alone” or not), work status (“work full-time,” “work part-time,” or “not working”), perceived health (5-point Likert scale ranging from “poor” to “excellent”), height, and weight were self-reported. BMI was calculated as kg/m^2^. These data were collected on day 0, before wearing the devices.

#### Physical Activity in Free-Living Conditions

Physical activity data were collected for 7 consecutive days (from day 1 to day 7). Participants were asked to simultaneously wear a TDKsw on their nondominant wrist (using an original nylon band) and an AG on the left side of the waist (using an elastic belt) during waking hours, removing only for water-based activities (eg, showering, bathing, or swimming) or contact sports.

The TDKsw uses signals from an embedded triaxial acceleration sensor to provide proprietary processed minute-by-minute physical activity data in CSV format. In this study, we focused on 2 outputs: METs and step count. The TDKsw is a small (device body size=52 mm×24.5 mm×13.5 mm), light (weight=26 g, including band), and rechargeable wristband-type device embedded with multiple sensors for health monitoring [9], and it can store minute-by-minute data for up to approximately 30 days. In this study, a dummy account was created for each participant, and the participant’s smartphone was used as a gateway to send the data to the cloud server, where the TDKsw data were downloaded. To handle missing data on the server, such as that caused by a communication error, the SilmeePro Wx software (version 2.1.0.0; TDK Corporation) was used to download the data from the device when needed.

The waist-worn AG was used as a reference to assess physical activity in free-living conditions because it is a valid and reliable monitor for estimating METs [13] and step count [14,15]. The AG is a research-grade triaxial accelerometer-based activity monitor (size=46 mm×33 mm×15 mm and weight=19 g); both raw and proprietary processed data can be downloaded using dedicated software. In this study, 1-minute epoch data on activity count and step count were downloaded in CSV format, using ActiLife software (version 6.13.4; ActiGraph LLC), in order to match data output from the TDKsw. Because the ActiLife algorithm uses only the vertical axis data in determining steps taken [16,17], the waist, which is near the center of mass, was considered more appropriate than the wrist to prevent false-positive step counts due to nonambulatory wrist movement under free-living conditions [16,18].

### Activity Monitor Data Processing

The data from the TDKsw and the AG were merged using the participant ID and the timestamp as keys. In doing this, records (minute-by-minute observations) were excluded if there were any missing values (eg, due to TDKsw device malfunction) or if the AG data were classified as nonwear [19,20]. There was no validated algorithm for TDKsw to identify nonwear; therefore, it was deemed that the participants followed the instruction to wear or remove both AG and TDKsw simultaneously. A valid day was defined as having ≥10 hours of accumulated AG wear time (convention for compliant wear time) [21] with an accumulation of ≥100 and <50,000 steps (for exclusion of outliers) [22] from both AG and TDKsw.

Data from each device were aggregated to daily step counts and time spent in MVPA (≥3 METs for the TDKsw and ≥1952 vertical axis activity counts/minute for the AG [13]) for each participant. Also, each valid day was classified as either an active day or an inactive day, using the data of step counts and time spent in MVPA from each device; valid days with ≥10,000 steps/day [23,24] or ≥21.4 minutes MVPA per day (corresponds to ≥150 minutes MVPA per week [5]) were classified as active days.

### Statistical Analysis

Baseline characteristic data were summarized as mean (SD) for continuous variables and as frequency and percentage for categorical variables.

The dataset in this study had a hierarchical structure, with daily aggregated data (maximum 7 valid daily observations per participant) nested within individuals. Therefore, multilevel models were used to appropriately account for this clustering. An intercept-only multilevel model was employed to estimate the overall mean (SE) of step counts and time spent in MVPA. Bland-Altman plots [25] were used to visualize the agreement between the TDKsw and the AG for each measure across each day where valid data were available. An intercept-only multilevel model was also applied to estimate the overall mean of the paired differences between the devices (ie, fixed bias) and the 95% limits of agreement (LoA) [26-28]. Proportional bias was evaluated based on the slope parameter of a multilevel model that included the average value between the devices for each outcome as a fixed effect.

To address potential model misspecification, robust inference was applied to all multilevel model analyses [29,30]. In addition, residual normality and homoscedasticity were assessed using diagnostic analyses for the Bland-Altman analysis models.

The proportion of agreement in classification as active day or inactive day was assessed using κ statistics. For interpretation of the κ statistics, we followed the ratings suggested by McHugh [31]: none (0.00‐0.20), minimal (0.21‐0.39), weak (0.40‐0.59), moderate (0.60‐0.79), strong (0.80‐0.90), and almost perfect (above 0.90).

Statistical analyses were performed using SPSS 29.0.1.0 (IBM Corporation) and R (version 4.5.0; R Foundation for Statistical Computing). The *lme4* and *clubSandwich* packages in R were used for multilevel modeling [32,33].

## Results

All 130 recruited participants agreed to be enrolled in the study and completed data collection. Of these, 1 participant was excluded from the analysis due to unexpected TDKsw malfunction attributable to the device or the user (only 371 out of 10,080 minutes of data were downloaded). Given this substantial data loss, we compared the data downloaded from the server with those extracted directly from each device using the SilmeePro Wx software. As a result, missing data in 27 participants due to communication errors were fully recovered using the software, while 35 participants had unrecoverable missing data caused by device malfunction or human error. Nevertheless, these 35 participants retained at least 92.6% of data during the measurement period. The final sample included 129 participants aged 23-89 years, of whom 51.2% (n=66) were women. Table 1 shows the full details of the participants’ characteristics.

**Table 1.** Descriptive characteristics of the participants.

Characteristics		Overall (N=129)	Men (n=63)	Women (n=66)
Age (years), mean (SD)		53.5 (18.9)	52.3 (18.5)	54.6 (19.3)
Age group (years), n (%)				
20‐29		16 (12.4)	6 (9.5)	10 (15.2)
30‐39		21 (16.3)	14 (22.2)	7 (10.6)
40‐49		20 (15.5)	10 (15.9)	10 (15.2)
50‐59		23 (17.8)	13 (20.6)	10 (15.2)
60‐69		14 (10.9)	4 (6.3)	10 (15.2)
70‐79		22 (17.1)	9 (14.3)	13 (19.7)
80‐89		13 (10.1)	7 (11.1)	6 (9.1)
Living area, n (%)				
National Capital Region		59 (45.7)	27 (42.9)	32 (48.5)
Other		70 (54.3)	36 (57.1)	34 (51.5)
Living arrangement, n (%)				
Living alone		36 (27.9)	11 (17.5)	25 (37.9)
Not living alone		93 (72.1)	52 (82.5)	41 (62.1)
Work status, n (%)				
Work full-time		80 (62)	45 (71.4)	35 (53)
Work part-time		20 (15.5)	7 (11.1)	13 (19.7)
Not working		29 (22.5)	11 (17.5)	18 (27.3)
Perceived health, n (%)				
Excellent		23 (17.8)	12 (19)	11 (16.7)
Very good		54 (41.9)	26 (41.3)	28 (42.4)
Good		52 (40.3)	25 (39.7)	27 (40.9)
Height (cm), mean (SD)		163.3 (9.4)	171.3 (5.1)	155.6 (5.4)
Weight (kg), mean (SD)		59.7 (11.4)	67.6 (9.1)	52.2 (7.7)
BMI (kg/m^2^), mean (SD)		22.3 (3.2)	23.0 (3.0)	21.6 (3.3)
BMI ≥25, n (%)		24 (18.6)	13 (20.6)	11 (16.7)

A total of 884 daily observations were used for the analysis (5 to 7 daily observations per participant). Estimated overall means of step counts and time spent in MVPA were 6369 (SE 242) steps/day and 40.3 (SE 1.9) minutes/day for the TDKsw, and 7572 (SE 237) steps/day and 34.8 (SE 1.9) min/day for the AG.

**Figure 2.**
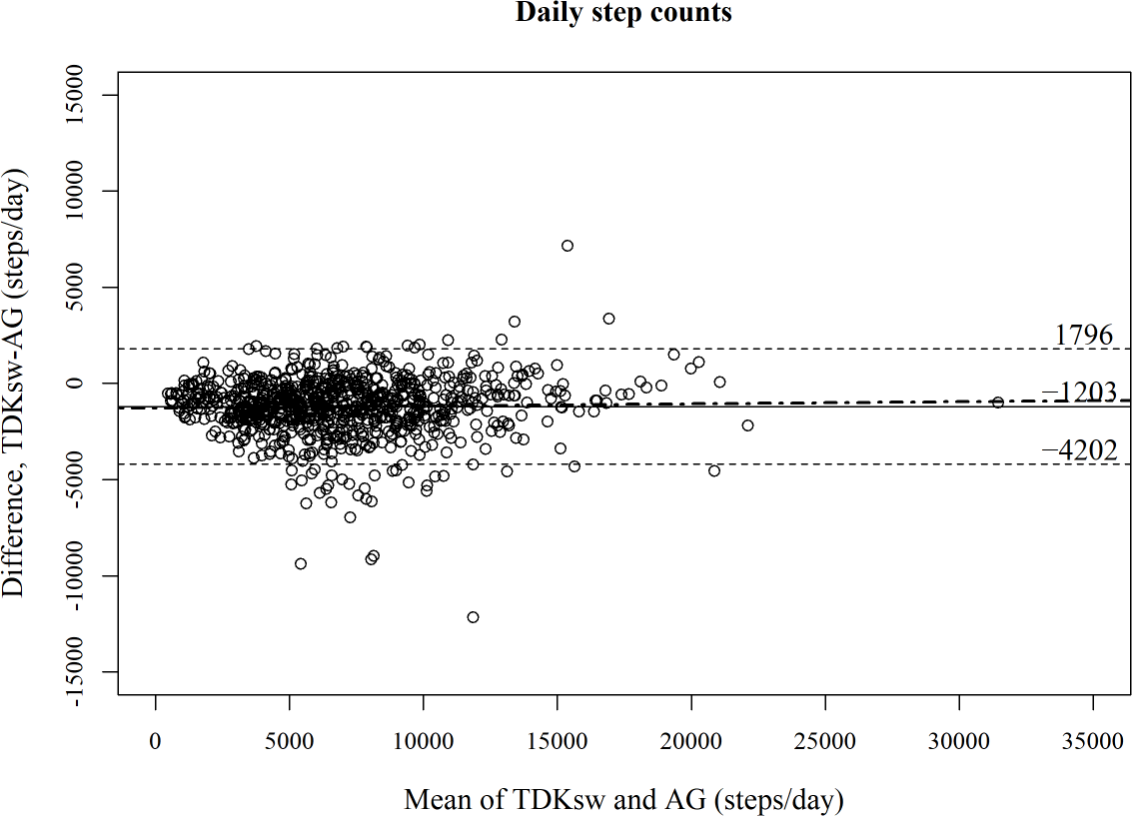
Bland-Altman plot comparing the TDK Silmee W22 to the ActiGraph wGT3X-BT for daily step counts (884 observations of 129 participants). The solid line represents the mean difference, the dashed lines represent the 95% limits of agreement, and the dot-dash line represents the linear regression lines by multilevel model. AG: ActiGraph wGT3X-BT; TDKsw: TDK Silmee W 22.

**Figure 3.**
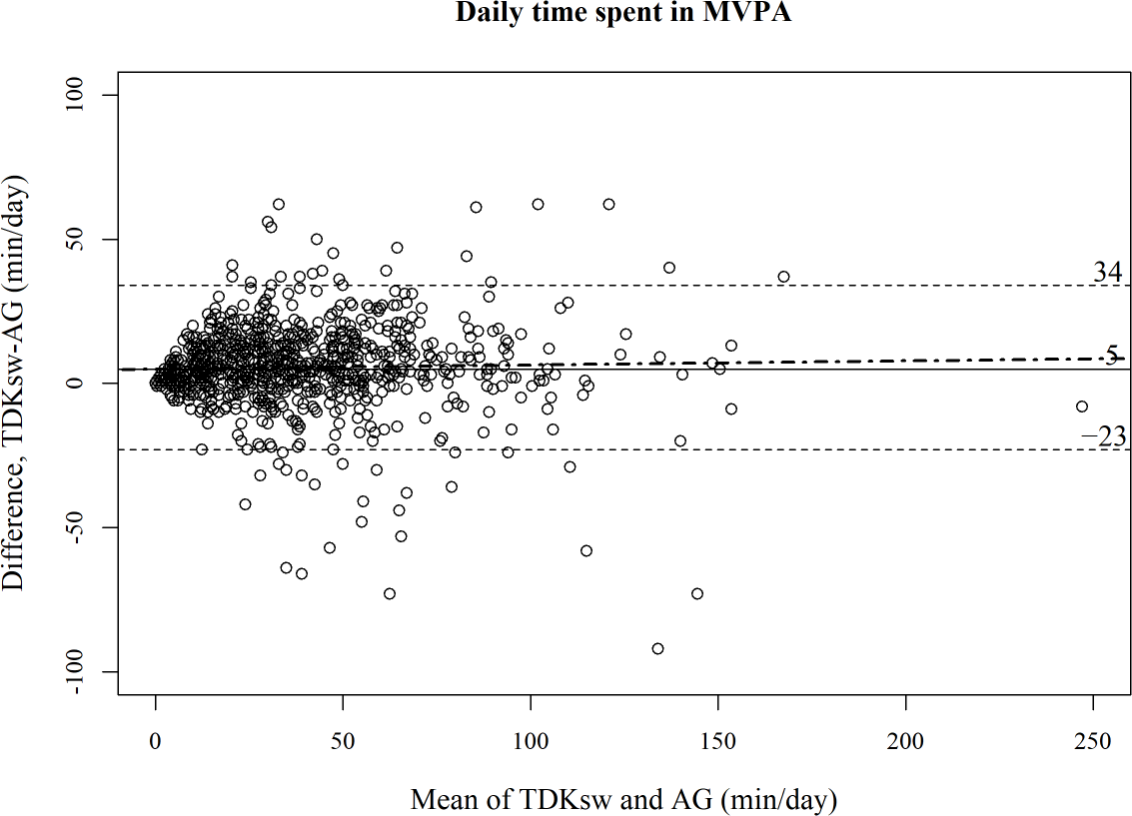
Bland-Altman plot comparing the TDK Silmee W22 to the ActiGraph wGT3X-BT for daily time spent in MVPA (884 observations of 129 participants). The solid line represents the mean difference, the dashed lines represent the 95% limits of agreement, and the dot-dash line represents the linear regression lines by multilevel model. AG: ActiGraph wGT3X-BT; TDKsw: TDK Silmee W 22; MVPA: moderate to vigorous physical activity.

Figures2  3 show Bland-Altman plots comparing TDKsw and AG for each metric. The Bland-Altman plots suggested no obvious proportional bias, as the regression slopes were not significant for step counts (mean 0.010, SE 0.017; *P*=.53) and MVPA (mean 0.014, SE 0.034; *P*=.676). Meanwhile, fixed biases were observed; the TDKsw estimated 1203 fewer steps per day (LoA −4202 to 1796; see Figure 2) and 5 more minutes per day (LoA −23 to 34) spent in MVPA (see Figure 3) compared with those estimated by the AG.

Multimedia Appendix 1 presents the results of residual normality and homoscedasticity diagnostics. Although the distribution of residuals appeared slightly heavy-tailed at both ends, no substantial skewness was observed in either direction. In addition, the residual versus fitted plots did not show any clear pattern of heteroscedasticity, with the spread of residuals remaining approximately constant across the range of fitted values. Based on these results, we considered it acceptable to interpret the Bland-Altman metrics under the assumptions of approximate normality and homoscedasticity.

Tables2  3 show the number of days classified as active or inactive by each device. The TDKsw and AG respectively classified 14% (124/884) and 23.1% (204/884) as active by daily step counts (see Table 2), and 70.4% (622/884) and 60.9% (538/884) by daily time spent in MVPA (see Table 3). The κ statistic was 0.65 (95% CI 0.59‐0.72) when classified by daily step counts and was 0.64 (95% CI 0.59‐0.69) when classified by daily time spent in MVPA, both indicating moderate agreement.

**Table 2.** Number of days classified as active or inactive by the TDK Silmee W22 and ActiGraph wGT3X-BT, based on daily step counts (884 observations of 129 participants).

		Days classified by AG^b^, n		
Days classified by TDKsw^a^, n		Inactive	Active^c^	Total
Inactive		673	87	760
Active^c^		7	117	124
Total		680	204	884

a TDKsw: TDK Silmee W22.

b AG: ActiGraph wGT3X-BT.

c Valid days with ≥10,000 steps/day were classified as active.

**Table 3.** Number of days classified as active or inactive by the TDK Silmee W22 and ActiGraph wGT3X-BT, based on daily time spent in moderate to vigorous physical activity (MVPA) (884 observations of 129 participants).

		Days classified by AG^b^, n		
Days classified by TDKsw^a^, n		Inactive	Active^c^	Total
Inactive		231	31	262
Active^c^		115	507	622
Total		346	538	884

a TDKsw: TDK Silmee W22.

b AG: ActiGraph wGT3X-BT.

c Valid days with ≥21.4 minutes MVPA per day were classified as active.

## Discussion

### Principal Findings

This study assessed the agreement between a wristband-type wearable IoT device, the TDKsw, and a research-grade activity monitor, the AG, worn on the waist, in estimating the daily accumulation of step counts and time spent in MVPA. Agreement was investigated at the day level under free-living conditions in young to older adults. Our results showed some discrepancies between the devices. Compared with the AG, the TDKsw underestimated step counts by approximately 1200 steps per day, whereas it overestimated the time spent in MVPA by 5 minutes per day. These differences seemed to be mostly constant over the measurement range. In addition, the results indicated moderate agreement in classifying whether a given person was active or not on that day; the levels of agreement were similar whether we used the data of daily step count or time spent in MVPA.

Our findings seemed to be counterintuitive, given that the daily step counts are expected to increase as people spend more time in MVPA [34]. A possible explanation for why step counts were underestimated, whereas MVPA was overestimated in this study, is that the MVPA measured by the TDKsw was not explicitly identified as walking. However, the exact reason remains unclear because this study used data that were preprocessed by the manufacturers’ proprietary algorithms.

The differences between the two devices are not trivial, equivalent to approximately 12 minutes of walking per day (assuming 100 steps per minute [35]) and one-fifth of the recommended level of physical activity (150 minutes of MVPA per week [5]). In addition, our results may have important implications for promoting physical activity. That is, some TDKsw users may be identified as active on that day even if in fact they are not, and vice versa. Such misclassification, which stems from the fixed bias observed in step counts and MVPA estimates, may be considered unacceptable for research or clinical purposes when assessing the amount or intensity of physical activity at a given point in time. However, when examining within-individual fluctuations of physical activity behavior over time, such differences would not substantially affect the interpretation of the data. The key advantage of the TDKsw is that it enables long-term monitoring and remote collection of large amounts of data by using IoT technology.

Although the κ statistics indicated moderate agreement, whether this level is acceptable depends on the intended use. As noted above, it may be insufficient for point-in-time assessments in clinical or research settings, but potentially acceptable for tracking temporal trends within individuals.

### Comparison With Previous Work

Comparing the present results with other free-living studies was challenging because there were differences in terms of methodology and the devices used. The only study to date that has investigated the accuracy of the TDKsw is Kimura et al [11], which reported a significant correlation (*r*=0.987; *P*<.001) between TDKsw step count and 9 minutes of video observation in older adults (n=20). Other related studies include research on the agreement between wristband-type activity trackers and waist-worn research-grade devices on a day-to-day basis. Chu et al [36] (n=35 males and n=69 females; median 31.0, IQR 26.0‐42.8 years; 7 days) reported that the Fitbit Flex overestimated steps/day relative to the waist-worn AG (median difference 1300 steps/day). Mikkelsen et al [37] (n=10 males and n=31 females; mean 47.6, SD 10.4 years; 7 days) reported that compared with the waist-worn ActiGraph GT3X, the Fitbit Charge 2 measured 1492 more steps per day (LoA −2250 to 5234), and 31 fewer MVPA minutes per day (LoA −132 to 71) with increasing difference as time in MVPA increased, suggesting that counterintuitive results might be obtained, as observed in our study. It should be noted that there was variability among the studies in the choice of ActiGraph count data (vertical axis or vector magnitude) and data-processing algorithms, which might have contributed to the mixed findings.

### Strength and Limitations

This study has several strengths. First, it included a relatively large sample of participants with a wide range of ages, equal gender balance, and various sociodemographic backgrounds. Second, the study was conducted under free-living conditions, which improved the ecological validity and the generalizability of the findings to real-life settings [38]. Third, we kept the observations (ie, daily aggregated data) as repeated measures for each participant and used multilevel analysis to calculate the LoA. This provides a more accurate representation of the actual agreement by lowering the risk of producing LoA that is too narrow [26]. Earlier agreement studies have rarely used multilevel analysis, despite the nested structure of the repeated measurement [27,37].

The major limitation of this study is the use of non–gold standard methods for comparing steps and MVPA. Having said that, there is no feasible gold standard method for assessing these measures in free-living conditions. In this study, we regarded the waist-worn AG as the best reference because the algorithms it uses to estimate METs and step count have been validated [13-15] and frequently used in earlier studies. Furthermore, although we instructed the participants to wear and remove both TDKsw and AG at exactly the same time, there is no way to ensure they followed this instruction. In addition, because the algorithms used in the TDKsw are proprietary and not publicly available, we were unable to provide a direct comparison of the algorithms.

### Conclusions

Differences were found in estimating the daily accumulation of step counts and time spent in MVPA when comparing outputs between the wrist-worn TDKsw and the research-grade waist-worn AG. It was noted that the TDKsw underestimated step counts and overestimated the time spent in MVPA, which might lead to misclassification of active days and inactive days. Wearable IoT devices such as the TDKsw are expected to be used for large-scale epidemiological studies and for promoting physical activity through long-term monitoring. When using TDKsw data over relatively short periods, caution is warranted, as misclassification—particularly when compared with data from research-grade monitors such as the ActiGraph—may affect feedback or goal-setting. Adjustments to classification thresholds may be necessary depending on the intended application.

## Supplementary material

10.2196/64602 Multimedia Appendix 1 Results of residual normality and homoscedasticity diagnostics.

## Abbreviations

AG: ActiGraph wGT3X-BT
IoT: Internet of Things
LoA: limits of agreement
METs: metabolic equivalents
MVPA: moderate to vigorous physical activity
TDKsw: TDK Silmee W22
