# Supplementary material for: Agreement Between TDK Silmee W22 and ActiGraph wGT3X-BT for Estimating Daily Step Counts and Moderate to Vigorous Physical Activity in Free-Living Adults: Comparative Study
Source: JMIR Form Res. 2025 Jul 30;9:e64602. doi: 10.2196/64602 (PMC12310187; doi:10.2196/64602)
Supplement: Multimedia Appendix 1 [file formative-v9-e64602-s001.doc]

Results of residual normality and homoscedasticity diagnostics.


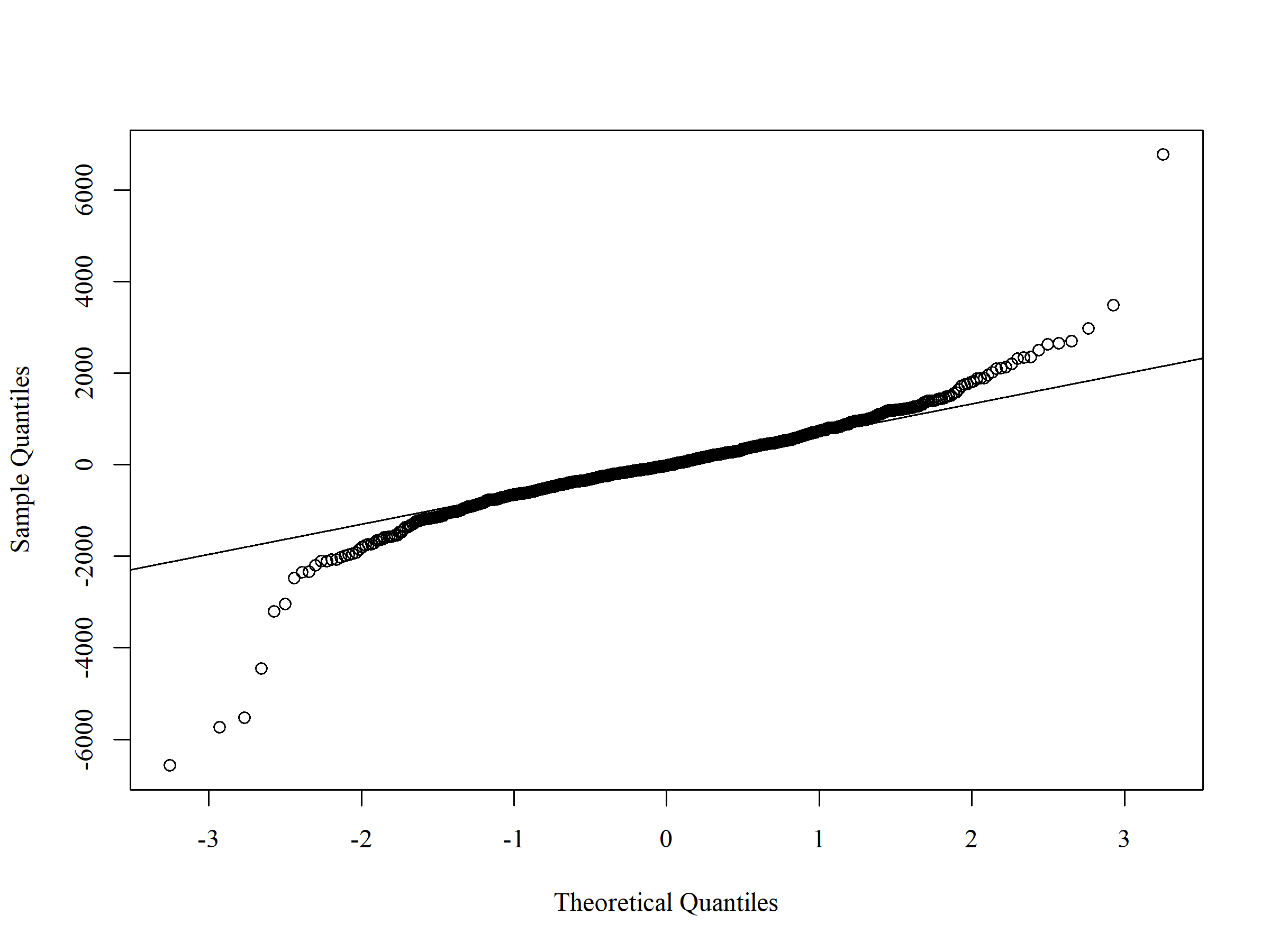

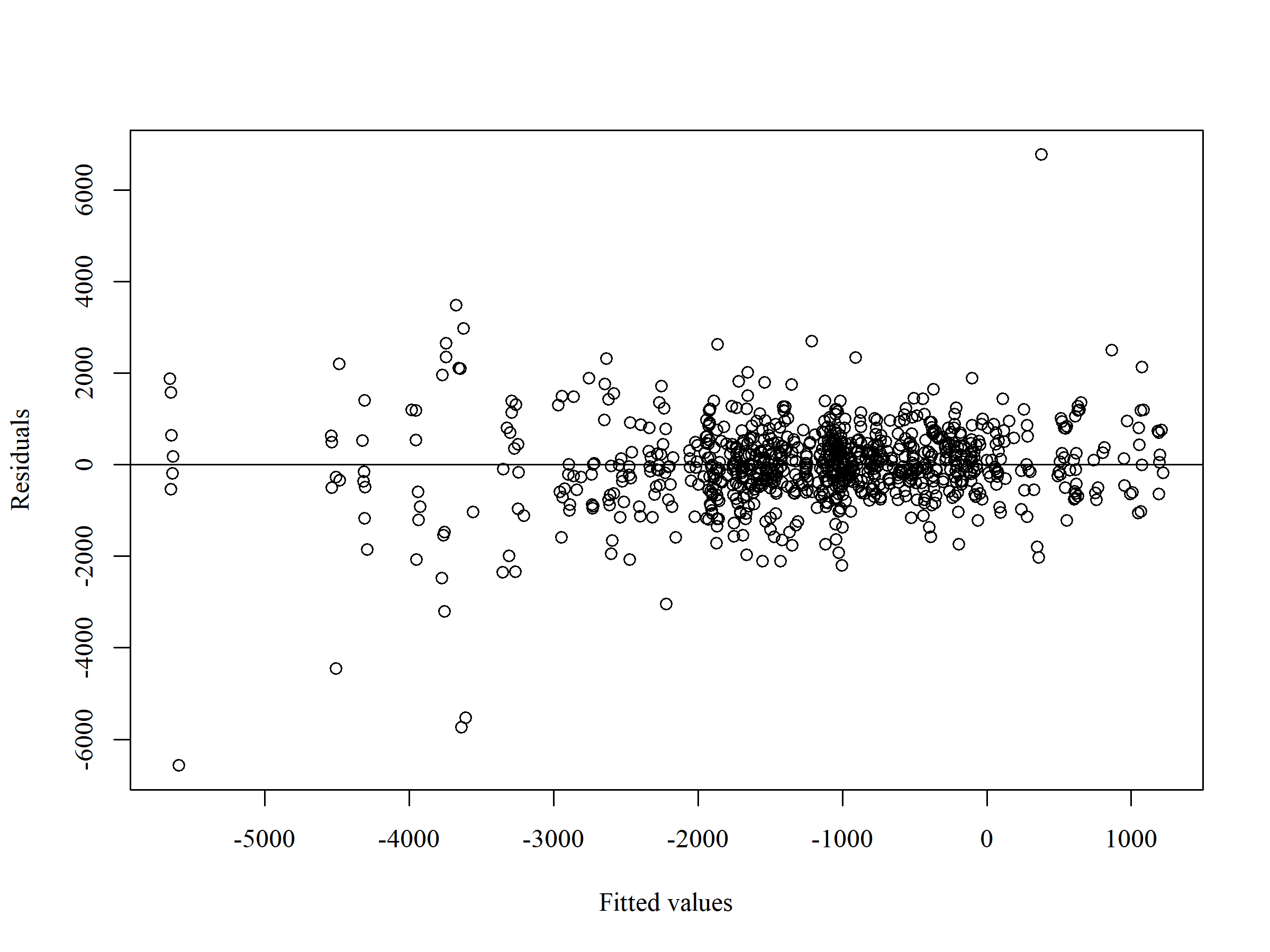


Figure S1. Diagnostic plots for residual normality and homoscedasticity in the Bland-Altman analysis models for daily step counts.

The left panel shows a normal quantile-quantile plot, and the right panel shows a plot of residual versus fitted values.


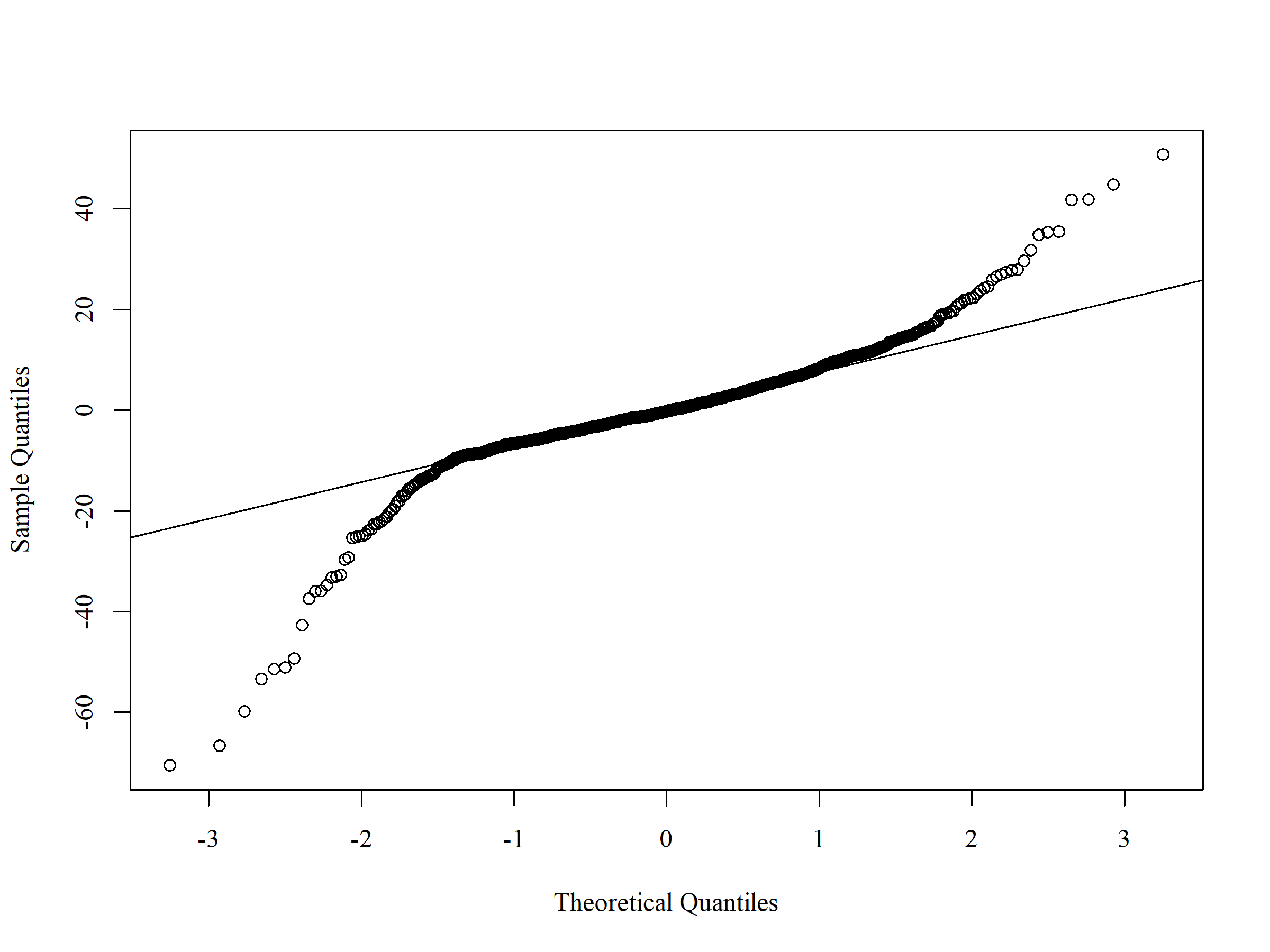

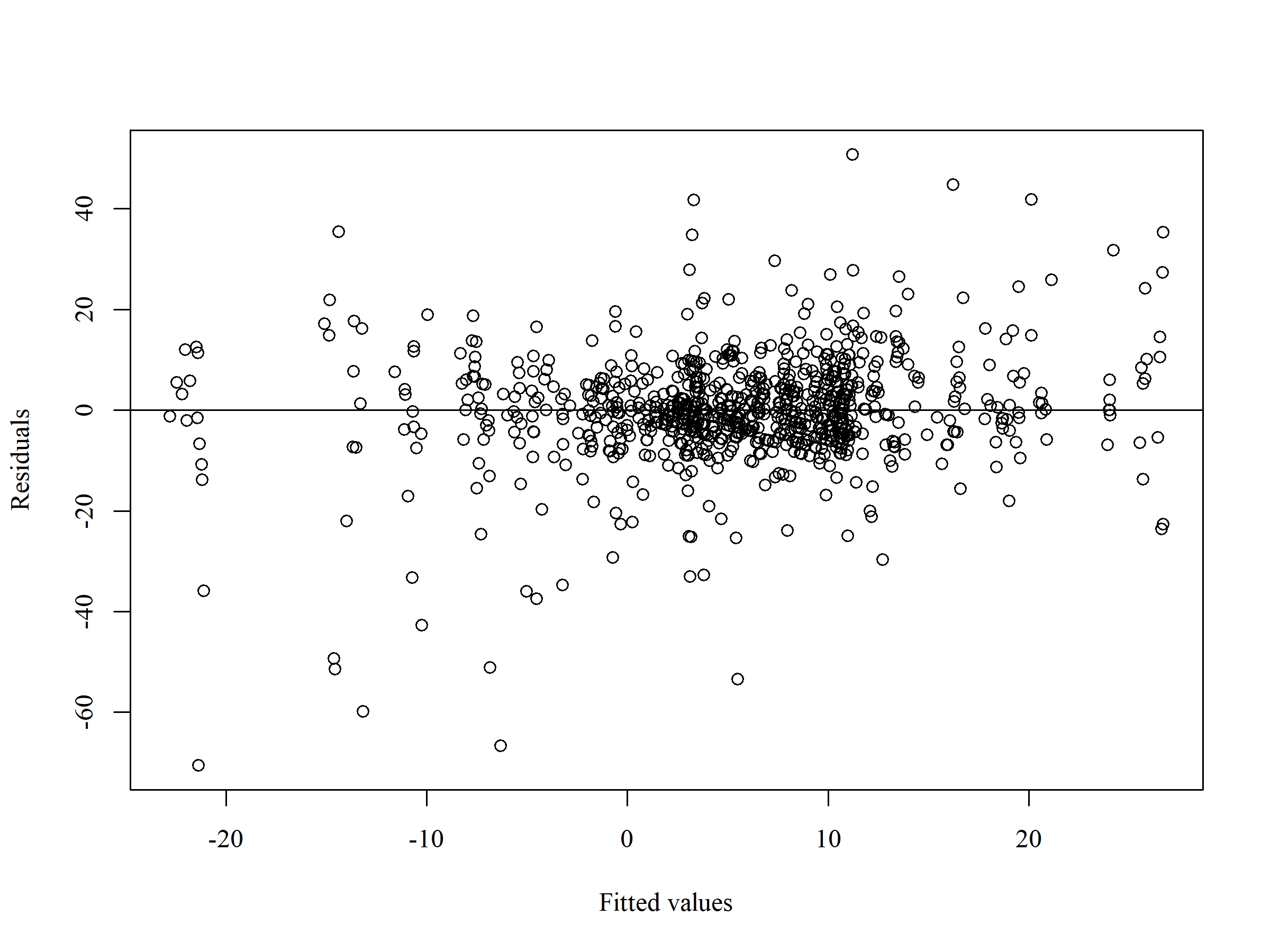


Figure S2. Diagnostic plots for residual normality and homoscedasticity in the Bland-Altman analysis models for daily time spent in MVPA.

The left panel shows a normal quantile-quantile plot, and the right panel shows a plot of residual versus fitted values. MVPA: moderate to vigorous physical activity.
